# Clinical inertia in newly diagnosed type 2 diabetes mellitus among patients attending selected healthcare institutions in Colombia

**DOI:** 10.1186/s13098-023-01245-0

**Published:** 2024-02-15

**Authors:** Nelson Alvis-Guzman, Martín Romero, Fernando Salcedo-Mejia, Maria Carrasquilla-Sotomayor, Lina Gómez, Mónica María Rojas, Juan Camilo Urrego, Claudia Catalina Beltrán, Jaime Enrique Ruíz, Adriana Velásquez, Juan Carlos Orengo, Adolfo Pinzón

**Affiliations:** 1 Universidad de la Costa, Cl. 58 #55 - 66, Barranquilla, Atlántico, Colombia; 2 Grupo de Investigación en Economía de la Salud, Universidad de Cartagena, Cartagena, Colombia; 3 Proyéctame Group, Bogotá, Colombia; 4 School of Public Health, Postgraduate Program in Epidemiology, Laboratory of Causal Inference in Epidemiology (LINCE-USP), University of São Paulo, São Paulo-SP, Brazil; 5 MSD Colombia, Bogotá, Colombia; 6 Merck, San Juan de Puerto, Puerto Rico; 7 Organon Chile, Santiago de Chile, Chile

**Keywords:** Clinical inertia, Glycaemic control, Type 2 diabetes mellitus, Adult

## Abstract

**Background:**

The burden of disease of diabetes in Colombia have increased in the last decades. Secondary prevention is crucial for diabetes control. Many patients already treated remain with poor glycemic control and without timely and appropriate treatment intensification. This has been called in the literature as *Clinical Inertia*. Updated information regarding clinical inertia based on the Colombian diabetes treatment guidelines is needed.

**Objective:**

To measure the prevalence of clinical inertia in newly diagnosed Type 2 Diabetes Mellitus (T2DM) patients in healthcare institutions in Colombia, based on the recommendations of the current official guidelines.

**Methods:**

An observational and retrospective cohort study based on databases of two Health Medical Organizations (HMOs) in Colombia (one from subsidized regimen and one from contributory regimen) was conducted. Descriptive analysis was performed to summarize demographic and clinical information. Chi-square tests were used to assess associations between variables of interest.

**Results:**

A total of 616 patients with T2DM (308 for each regimen) were included. Median age was 61 years. Overall clinical inertia was 93.5% (87.0% in contributory regimen and 100% in subsidized regimen). Patients with Hb1Ac ≥ 8% in the subsidized regimen were more likely to receive monotherapy than patients in the contributory regimen (OR 2.33; 95% CI 1.41–3.86).

**Conclusions:**

In this study, the prevalence of overall clinical inertia was higher in the subsidized regime than in the contributory regime (100% vs 87%). Great efforts have been made to equalize the coverage between the two systems, but this finding is worrisome with respect to the difference in quality of the health care provided to these two populations. This information may help payers and clinicians to streamline strategies for reducing clinical inertia and improve patient outcomes.

## Introduction

Diabetes mellitus (DM) is a chronic condition that affects an important proportion of the population around the world. The Institute of Health Metrics and Evaluation (IHME), estimated that 529 million (95% uncertainty interval [UI] 500–564) people living with diabetes worldwide, and the global age-standardised total diabetes prevalence was 6.1% (5.8–6.5) (1). Globally in 2021, there were 37.8 million (95%CI 35.4–40.2) of Years of Life Lost (YLL) and the global age-standardised rate of YLL per 100,000 due to diabetes of 437.4 (409.2–464.1); in Colombia there were 157 thousand YLL (134–184) and a rate of 279.9 (239.2–328.2) [1].

Several factors contribute with the increasing number of patients, as population aging, economic development and higher urbanization, that together lead to sedentary lifestyles, more consumption of unhealthy food and obesity [2]. Meanwhile, countries are working on the implementation of strategies for early detection and more effective treatment that also contribute to this rising prevalence. In Colombia, the prevalent and incident cases have been increasing during the last years due to the measures taken to enhance the notification and registry in diabetes programs, of both subsidized (health insurance for unemployed people) and contributory (health insurance for people with payment capacity) regimens. According to the “Cuenta de Alto Costo” (an institution of the Colombian health system that keeps the registry of high-cost diseases that occur in the country based on the reports of all Health Medical Organizations [HMOs]) [3] during the last period of study (July 1st2020 to June 30th2021) there were 1,576,508 patients with diabetes mellitus that account for a prevalence of 3.11 cases per 100 inhabitants. This value contrasts with other publications were the prevalence estimated for the country ranges between 7 and 9% [4, 5].

The implementation of strategies for an effective control of T2DM patients is fundamental to reduce microvascular and macrovascular complications associated with T2DM, improve quality of live, and decrease the costs associated.

Despite clinical advances in the management of T2DM, it has been reported that nearly 48% of the patients with T2DM do not achieve the recommended glycemic goal of HbA1c < 7% [5]. Moreover, many patients already treated remain with poor glycemic control and don´t receive timely and appropriate intensification of therapy. This phenomenon has been called *Clinical Inertia*, defined as failure by healthcare providers to initiate or intensify treatment in a timely manner [6].

Reasons for clinical inertia in T2DM include lack of time and resources to address patient problems, overestimation of care provided, lack of communication between healthcare professionals and patients, non-adherence to medications, attitudes and beliefs of patients and community/cultural issues, among others [6]. The negative impact of clinical inertia on glycemic control has been reported in several studies [7–10], so monitoring and controlling the extent of clinical inertia in clinical practice could be crucial in reducing the healthcare burden associated with T2DM.

**Fig. 1:**
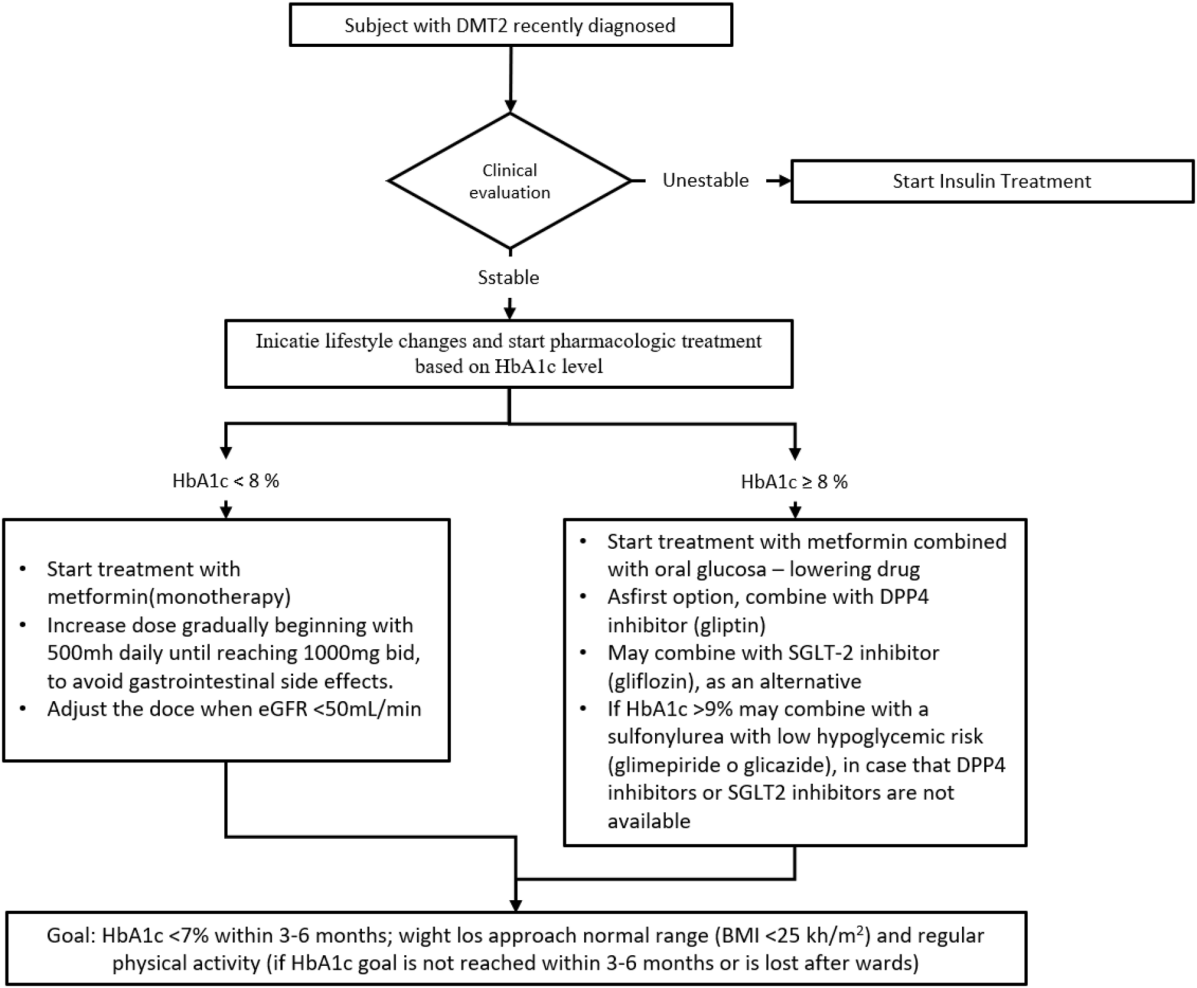
Algorithm of initial pharmacological approach based on HbA1c cut-off points. Adapted from Colombian diabetes guideline

The Colombian diabetes treatment guidelines “Guía de práctica clínica para el diagnóstico, tratamiento y seguimiento de la diabetes mellitus tipo 2 en la población mayor de 18 años” (CPG) were published in 2016 by the Ministry of Health [11]. The purpose of this guideline is to recommend the best clinical practice, based on strong evidence, regarding screening, diagnosis, initial treatment, control, and detection of complications of T2DM for physicians. The guideline should be used by physicians on HMOs (see Fig. 1).

In Colombia, to our knowledge, there is only two published studies [12, 13] that have measured clinical inertia in T2DM patients. Although these are representative studies, each one of them assessed only patients from one of the regimens (contributive or subsidized), so they did not show a complete picture of the Colombian population. In addition, one study was developed during 2015 and used ADA guidelines as a reference to measure clinical inertia, meanwhile Colombian National Guidelines were published in 2016. Thus, updated information regarding clinical inertia and the associated factors using more recent data may help healthcare policymakers and clinicians streamline strategies for reducing clinical inertia in T2DM and improve patient outcomes.

In this study we aim to measure the prevalence of clinical inertia in newly diagnosed Type 2 Diabetes Mellitus patients in healthcare institutions in Colombia from the subsidized and contributory regimen, based on the recommendations of the current official guidelines.

## Methods

### Design and study population

This was an observational and retrospective cohort study that was based on medical records included in databases of two Health Medical Organizations (HMOs) in Colombia, one from the subsidized regimen and one from contributive regimen. The inclusion period extended from 1 April 2016 to 31 March 2017, with a minimum follow-up of one year per patient from the inclusion date.

### Sample size

Determination of the required sample size was performed in accordance with some assumptions. The number of newly diagnosed T2DM patients in Colombia was 93.642 in 2016, according to the *Cuenta de Alto Costo (CAC)*. The average prevalence of clinical inertia found in the literature was 30% [14], so considering a 95% confidence interval and 4% error, the sample size calculated was 604 patients. Each HMO recruited half of the patients since the distribution of affiliation to each insurance regimen in Colombia is around 50%. The selection of the samples was made in a probabilistic way.

The Ethics Committee of the participants HMOs approved this research.

### Inclusion and exclusion criteria

Patients eligible for inclusion in this study were: (1) aged ≥ 18 years; (2) newly diagnosed Type 2 Diabetes Mellitus (T2DM) (Fasting blood glucose: ≥ 126 mg/d; or HbA1c ≥ 6.5%; or postprandial glucose ≥ 200 mg/dl) (International Classification of Diseases 10th Revision, Clinical Modification [ICD-10-CM] codes: E11.0–E11.9); (3) complete information in the databases regarding variables of interest. Patients were excluded from the study if they presented diagnosis of type 1 diabetes ([ICD-10-CM] codes: E10.0-E10.9) or gestational diabetes ([ICD-10-CM] codes: O24.0–O24.9).

### Study variables

Demographic and clinical variables: The demographic variables collected included age, sex, city of origin and HMO. The clinical variables included were height, weight, BMI, history of hypertension, dyslipidemia, heart attack, cerebrovascular disease, chronic kidney disease and tobacco use. These records were obtained for each patient at the index date. Also, we collected information about date of diagnosis, HbA1c at diagnosis, time from diagnosis to treatment initiation, and HbA1c at follow up (3–6 months).

Medication variables: Class of treatment prescribed at diagnosis (monotherapy, dual therapy and triple therapy), therapeutic group at diagnosis (metformin, sulfonylureas, dipeptidyl peptidase 4 inhibitors (iDDP-4), ultra-rapid acting insulin, low-acting insulin, intermediate-acting insulin), treatment adjustment and type of medication added. The Fig. 1, show the algorithm of initial pharmacological approach based on HbA1c cut-off points. Adapted from Colombian diabetes guideline.

Overall clinical Inertia: was defined as failure to initiate or intensify therapy in a timely manner (at diagnosis and/or 3–6 months after diagnosis) according to local evidence-based “Clinical National Guide (CNG) for diagnosis, treatment and follow-up of T2DM in population over 18 years old” of Colombia. Clinical Inertia was also analyzed by Initial Inertia and Follow-up Inertia.

Initial Inertia: was defined as failure to initiate the recommended pharmacological treatment according to CNG or to a late dispensing (> 30 days) of it.

Follow-up Inertia: was defined as a failure to adjust therapy at 3–6 months after diagnosis (considering Hb1Ac level or the lack/delay in testing) according to CNG.

### Statistical analysis

Descriptive analysis was carried out. Categorical variables were described with absolute and relative frequencies, and numerical variables were described as median and interquartile range with 95% confidence intervals (95% CI). The Chi-square test was used for comparisons of categorical variables. Two-sided p values of less than 0.05 were considered statistically significant. Analyses were conducted overall, in two subgroups according to Hb1Ac level (< 8% and ≥ 8%) and by insurance regimen (subsidized and contributive). All analyses were conducted using STATA 14.

## Results

### Demographic and clinical characteristics

We identified 616 patients with a new diagnosis of T2DM (308 for each regimen). The median age was 61 (range: 20–96 years) and almost 65% (n = 399) were women. From a total of 19 departments, Bolívar, Atlántico and Valle del Cauca had the highest representativeness, with 19.8% (n = 122), 18.7% (n = 115) and 15.7% (n = 97); respectively. The demographic and clinical characteristics are shown in Table 1.

**Table 1:** Demographic and clinical characteristics of newly diagnosed T2DM patients

Variables	Number (%)
Demographics	
Sex	
Male	217 (35.2)
Female	399 (64.8)
Age	
< 60 years	298 (48.4)
≥ 60 years	318 (51.6)
Type of insurance regimen	
Contributory	308 (50.0)
Subsidized	308 (50.0)
Clinical	
BMI	
Underweight	12 (1.9)
Normal	158 (25.6)
Overweight	258 (41.9)
Obesity	188 (30.5)
HbA1c at diagnosis	
< 8%	331 (53.7)
≥ 8%	285 (46.3)
Medical history	
Acute Myocardial Infarction	
Yes	23 (3.73)
No	593 (96.3)
Cerebrovascular disease	
Yes	22 (3.6)
No	594(96.4)
Smoking	
Yes	69 (11.2)
No	547 (88.8)
Comorbidities	
Hypertension	
Yes	443 (71.9)
No	173 (28.1)
Dyslipidemia	
Yes	299 (48.5)
No	317 (51.5)
Chronic kidney disease	
Yes	504 (81.8)
No	112 (18.2)
Chronic kidney disease stage	
Stage 1	190 (37.7)
Stage 2	168 (33.3)
Stage 3A	75 (14.9)
Stage 3B	52 (10.3)
Stage 4	11 (2.2)
Stage 5	1 (0.2)
No data	7 (1.4)

### Type of treatment dispensed at the time of diagnosis

Considering the HbA1c level at the time of diagnosis, it was found that 53.7% (n = 331) of the patients showed a HbA1c value < 8%. In this group, monotherapy was started in 87.9% (n = 291) of the cases, dual therapy in 11.2% (n = 37) and triple therapy in 0.9% (n = 3).

Regarding monotherapy, the most frequent medication formulated was metformin (88%), followed by slow-acting basal insulin (6.2%) and sulfonylureas (2.7%). In dual therapy, the most frequent combinations were metformin + sulfonylureas (40.5%), metformin + slow-acting basal insulin (37.8%), and ultra-fast-acting prandial insulin + slow-acting basal insulin (8.1%). Finally, in triple therapy, it was observed that the most frequent combination was Metformin + ultra-fast-acting prandial insulin + slow-acting basal insulin (66.7%) followed by the combination of Sulfonylureas + ultra-fast-acting prandial insulin + Slow-acting basal insulin (33.3%).

From the 285 patients who showed HbA1c ≥ 8%, 66% (n = 188) initiated treatment with monotherapy, 32.3% (n = 92) with dual therapy and 1.8% (n = 5) with triple therapy. In the same way as in patients with HbA1c < 8%, the most frequent medications formulated in monotherapy were metformin (69.7%), slow-acting basal insulin (20.2%), and sulfonylureas (3.7%). In dual therapy: metformin + sulfonylureas (31.5%), metformin + slow-acting basal insulin (37.8%), and ultra-fast-acting prandial insulin + slow-acting basal insulin (20.7%). In triple therapy: Metformin—Sulfonylureas—Slow-acting basal insulin (40%) and Metformin—Ultra-fast-acting prandial insulin—Slow-acting basal insulin (40%).

### Mono, dual, and triple therapy by insurance regimen

A comparison regarding the type of treatment prescribed in each insurance regimen according to Hb1Ac level at the time of diagnosis was performed. It was observed that more than 80% of the patients in both regimens with Hb1Ac < 8% were treated with monotherapy, following the national guideline recommendations. A different situation in patients with HbA1c ≥ 8% was noted. It was found that more than a half did not follow the national recommendations, starting with monotherapy and finding almost 20% more patients with this treatment in the subsidized regimen than in the contributory regimen (74.8% vs. 56%). Regarding the dual therapy -that is recommended for this group-, it was started in a greater proportion in the contributory regimen than in the subsidized (41.0% vs. 24.5%).

Finally, a stratified analysis was carried out by HbA1c level (< 8% and ≥ 8%) to evaluate if there were differences between the type of treatment initiated by insurance regimen. It was established that in patients with HbA1c < 8%, no statistical differences were observed in the type of treatment started in both regimens (OR 1.41; 95% CI 0.71–2.76). However, in patients with Hb1Ac ≥ 8%, statistical differences were observed, showing that patients from the subsidized regimen were 1.3 times more likely to receive monotherapy (contrary to the guideline) than patients from the contributory regimen (OR 2.33, 95% CI 1.41–3.86).

## Prevalence of clinical inertia

The overall clinical inertia for this study was 93.5% (n = 576). When it was analyzed by insurance regimen, it was observed that 87.0% (n = 268) of patients from the contributory regimen presented this outcome; while, this percentage was 100% (n = 308) for patients of the subsidized regimen. Subsequently, an analysis regarding type of inertia was carried out. It was found that 58.6% (n = 361) of patients presented initial inertia, being the proportion higher in the contributory than in the subsidized regimen (61.0% vs. 56.2%). From the 41.4% of patients who did not present initial inertia, 84.3% (n = 215) of them showed follow-up inertia, being 100% (n = 135) in the subsidized regimen (see Table 2).

**Table 2:** Type of clinical inertia among newly diagnosed T2DM patients

Type of inertia	Total	Contributory	Subsidized
	(n = 616)	(n = 308)	(n = 308)
	n (%)	n (%)	n (%)
Overall clinical inertia			
Yes	576 (93.5)	268 (87.0)	308 (100)
No	40 (6.5)	40 (13.0)	0 (0)
Initial Inertia			
Yes	361 (58.6)	188 (61.0)	173 (56.2)
No	255 (41.4)	120 (39.0)	135 (43.8)
Follow-up Inertia (no initial inertia)			
Yes	215 (84.3)	80 (66.7)	135 (100)
No	40 (15.7)	40 (33.3)	0 (0.0)

Inertia associated factors were studied and shown in Table 3. Regarding initial inertia, inadequate treatment was observed in 84.5% (n = 305) of the cases and a late beginning of treatment (supply of medication > 30 days) in 15.5% (n = 56) of the cases. When the results were discriminated by insurance regimen, it was found that all patients (n = 173) in the subsidized regimen received inadequate treatment, while this situation was 70.2% (n = 132) for the contributory regimen. Concerning associated factors for follow-up inertia, it was found that 93.0% (n = 200) of the cases were due to the lack or delay in follow-up HbA1c testing within the 3–6 months following diagnosis; while 7.0% (n = 15) were due to the lack of proper adjustment or inadequate adjustment of the treatment as per indicated by the follow-up HbA1c level. The 100% (n = 135) of the cases in the subsidized regimen showed lack or delay in follow-up HbA1c testing.

**Table 3:** Inertia associated factors in newly diagnosed T2DM patients

Inertia associated factors	Total (N = 576)	Contributory (N = 268)	Subsidized (N = 308)
	n (%)	n (%)	n (%)
Initial inertia	(n = 361)	(n = 188)	(n = 173)
Inadequate treatment	305 (84.5)	132 (70.2)	173 (100)
Late beginning of treatment (supply > 30 days)	56 (15.5)	56 (29.8)	0 (0.0)
Follow-up Inertia	(n = 215)	(n = 80)	(n = 135)
Inadequate treatment adjustment	15 (7)	15 (18.7)	0 (0.0)
No HbA1c within the 3–6 months	42 (19.5)	40 (50.0)	2 (1.5)
One HbA1c (at diagnosis)	158 (73.5)	25 (31.3)	133 (98.5)

### Analyses by main variables

#### A) Overall clinical Inertia and age

From the entire sample, 45.3% (n=279) of participants presented overall clinical inertia and were <60 years, while 48.2% (n=297) had overall clinical inertia but were ≥60 years. Not association was observed when analyzing this outcome by these age groups. (OR 0.76; 95% CI 0.40–1.46).

#### b) Overall clinical Inertia and obesity

From the entire sample, 26.9% (n=166) of participants showed overall clinical inertia and were obese, while 66.6% (n=410) had this outcome but were not obese. Statistical association was found between obesity and overall clinical inertia, observing that obese patients were 67% less likely to present overall clinical inertia (OR 0.33; 95% CI 0.17–0.63).

#### c) Overall Clinical Inertia and number of comorbidities

From the entire sample, 35.7% (n=220) of participants presented overall clinical inertia and ≤2 comorbidities, while 57.8% (n=356) had this outcome and >2 comorbidities. No association between the number of comorbidities grouped and overall clinical inertia was found (OR 1.85; 95% CI 0.88–3.86).

### Patients who reached the goals

To analyze the proportion of patients who achieved goals (HbA1c < 7%), 255 patients without initial inertia were identified. Only 55 (21.6%) of them had follow-up HbA1c testing within the following 3–6 months and 43 were in goals; that is, 16.9% of the patients without initial inertia had reached the objectives proposed by the national guideline. When analyzing this number based on the total number of patients who were included in the study, only 6.98% achieved the HbA1c goal proposed by the guideline.

## Discussion

The overall clinical inertia for this study was 93.5% (n = 576) with differences by insurance regimen (87.0% contributory regimen and 100% subsidized regimen). By type of inertia, 58.6% of patients presented initial inertia, being the proportion higher in the contributory than in the subsidized regimen (61.0% vs. 56.2%). From the 41.4% of patients who did not present initial inertia, 84.3% of them showed follow-up inertia. Unlike obesity, no association between the number of comorbidities grouped and age and overall clinical inertia was found.

In order to reduce the risk of both microvascular and macrovascular complications the importance of glucose control has been acknowledged [15–17]. Its importance has been extensively communicated through current national and international clinical guidelines [18, 19]. Nonetheless, several T2DM patients do not reach goals regarding glycemic control during the first year of diagnosis and do not receive proper treatment intensification [6, 20]. This failure of physicians to initiate or intensify therapy in a timely manner, despite recognition of the problem, has become known as clinical or therapeutic inertia.

Half of the patients in this study belong to the contributory regimen and the other half to the subsidized regimen which reflects the situation of insurance affiliation in the general population in the country. This is important because sometimes, assumptions around the quality of health services are made based on the type of insurance of the patients. In this study, the prevalence of overall clinical inertia was 93.5%, however it was higher in the subsidized regime than in the contributory regime (100% vs 87%). Great efforts have been made to equalize the coverage between the two systems, but this finding is worrisome with respect to the difference in quality of the health care provided to these two populations. The prevalence of clinical inertia was higher than those observed in other countries like United States, where it was found a prevalence around 70% [21], or in Spain where this value was 52% [22]. These differences may be related to geographic variation, as well as the methods of data collection.

Regarding type of inertia, 58.6% presented initial inertia. Immediate, intensive treatment for newly diagnosed patients may be necessary to avoid irremediable long-term risk for diabetic complications and mortality. It has been estimated that inadequate glycemic control is responsible for over 200,000 diabetes-related complications per year in North America alone, resulting in excess healthcare costs and tens of thousands of premature deaths [23, 24]. Among patients with newly diagnosed diabetes and 10 years of survival, HbA1c levels ≥ 6.5% for the 1st year after diagnosis were associated with worse outcomes [25].

It is noteworthy that more than 80% of the population without initial inertia, showed follow-up inertia and that this value was 100% for the subsidized regimen. This finding puts into consideration the need to evaluate not only the management of these patients, but also the effective access to the T2DM control programs. Finally, it is critical that 93% of the cases of follow-up inertia were related to the lack of HbA1c testing between 3 and 6 months after diagnosis. This highlights the difficulties faced in achieving strict follow-up of these patients (in order to control the disease) but at the same time, is consistent with what was observed in reports from other countries [26]_._

Comorbidities in type 2 diabetes patients are variables related to prognosis and are also an important consideration when a treatment is initiated. In this analysis the frequency of overweight or obesity (72.4%) were almost identical to another previous study in a Colombian population [13]_._ This study also found hypertension in 71,9%, dyslipidemia in 48.5% and chronic kidney disease in 81.8% of the patients, which shows a particularly different pattern when compared to other studies such as the review of 1,514,966 eligible patients with T2DM of the Quintiles Electronic Medical Record database that compared comorbidities in patients with and without CVD and found hypertension in 98% and 91%, hyperlipidemia in 95% and 79%, and chronic kidney disease in 39% and 19%; respectively [27]_._ It is worth noting that from the patients who had chronic renal disease, 25.2% of the patients were in stage 3, which for a population of newly diagnosed patients means that they arrive at the diagnosis late in the natural history of the disease. This is an important consideration both from the public health perspective but also from the clinical care of each individual patient.

In general, treatment of type 2 diabetes mellitus begins with lifestyle changes and oral antidiabetic drugs. In this study, monotherapy was selected in the 87.7% of the patients with HbA1c < 8%, and metformin was given in the 88% of these cases. In this sense, even though not all the patients were properly treated, a clear and important adherence to the National Guideline was observed. In Colombia, similar observations were made in 2017 by Machado et al. [12], who reported that metformin was used in the 84% of the cases, notwithstanding that this percentage includes the whole population under study.

On the other hand, when analyzing patients with HbA1c ≥ 8%, it was observed that two-thirds of this group were not adequately treated, since monotherapy was initiated at diagnosis. When comparing both regimens, we found that patients from the subsidized regimen were 1.3 times more likely to receive monotherapy than patients from the contributory regimen (OR 2.33, 95% CI 1.41–3.86). This raises concerns about the way in which the National Guideline is being adopted for the treatment of patients with the greatest risks of this pathology and inequities that may occur between regimes regarding the access to adequate therapy.

As described in the literature, here we identified a failure to initiate an adequate treatment^(6)^_._ It will be valid and necessary in further studies to explore if the cause of the inadequate management is due to factors directly related to the health care professionals, attributable to poor knowledge of the Guideline, failure to set clear goals, reactive more that proactive treatment, among others; or if it is more related to possible administrative barriers imposed by our health care system when prescribing multiple medications for control of diabetes.

There were some limitations. Analyses were carried out from an administrative database and not for medical charts, which not allow to confirm the causes of clinical inertia. Likewise given the source of the sample and the study design, the results are not generalizable for all the population in Colombia. The distribution was not proportional to the sizes of the population with T2DM attended in each department. Considering the high number of HMOs in Colombia, sample size in this study is another limitation we found to reflect the whole picture of patients with DM2.

It is important to highlight that at that time the information was collected (2016–2017), some medications for T2DM included in the CNG, were not covered by the health benefit plan (a list of services -including medications- that should be provided by the HMOs). Additionally, the subsidized regimen had at that time a different payment process (through charges to the local Health Secretary) than the contributory one (through direct charges to an account of the ministry of health), generating difficulties to the health care institutions for the prescription of medications not included in the health benefit plan in that moment.

The variations in payment conditions could have caused differences in the diagnosis, treatment, monitoring, and control of the disease between the regimens [11, 28]_._ However, the possibility that the differences in results observed in the two HMOs could be related to differences in data collection methods, cannot rule out.

Since this study found 93.5% of the population had clinical inertia, it is necessary to evaluate the correct implementation of current guidelines for the management of T2DM and their impact in terms of improvements of health conditions. Finally, it is necessary to increase the awareness of the physicians about clinical inertia and the consequences of the lack of the glycemic control (12).

Finally, in this study the non-pharmacological treatment were not considered, being this a possibility of bias.

## Conclusion

This is the first study that analyzes clinical inertia under the current local clinical guidelines in both regimens of insurance affiliation in Colombia. In this study, clinical inertia constitutes a major issue for the diabetic population, not only at the time of diagnosis, but also during the follow-up of the patients, particularly in the subsidized regimen. It is important to review the guidelines awareness, knowledge and adherence. This information may help payers and clinicians to streamline strategies for reducing clinical inertia and improve patient outcomes.

## Data Availability

The datasets generated and/or analyzed during the current study are not publicly available but are available from the corresponding author on reasonable request.
