# Correction: Clinical inertia in newly diagnosed type 2 diabetes mellitus among patients attendingselected healthcare institutions in Colombia

**DOI:** 10.1186/s13098-024-01300-4

**Published:** 2024-03-25

**Authors:** Nelson Alvis-Guzman, Martín Romero, Fernando Salcedo-Mejia, Maria Carrasquilla-Sotomayor, Lina Gomez, Mónica María Rojas, Juan Camilo Urrego, Claudia Catalina Beltrán, Jaime Enrique Ruíz, Adriana Velásquez, Juan Carlos Orengo, Adolfo Pinzón

**Affiliations:** 1 Universidad de la Costa, Cl. 58 #55 - 66, Barranquilla, Atlántico, Colombia; 2 Grupo de Investigación en Economía de la Salud, Universidad de Cartagena, Cartagena, Colombia; 3 Proyéctame Group, Bogotá, Colombia; 4 School of Public Health, Postgraduate Program in Epidemiology, Laboratory of Causal Inference in Epidemiology (LINCE-USP), University of São Paulo, São Paulo-SP, Brazil; 5 MSD Colombia, Bogotá, Colombia; 6 Merck, San Juan de Puerto, Puerto Rico; 7 Organon Chile, Santiago de Chile, Chile

**Correction:** ***Diabetology & Metabolic Syndrome (2024) 16:42***

10.1186/s13098-023-01245-0

The copyright holder for this article was incorrectly given as “Battelle Memorial Institute, under exclusive licence to Springer Nature Switzerland AG 2024” but should have been “© Merck & Co., Inc., Rahway, NJ, USA and its affiliates and Nelson Alvis- Guzman, Martín Romero, Fernando Salcedo-Mejia, Maria Carrasquilla- Sotomayor, Lina Gómez, Adolfo Pinzón 2024’.

The original article has been revised.

